# *Sirt1* deletion in hypertrophic chondrocytes differentially regulates skeletal growth and bone accrual in males and females mice

**DOI:** 10.3389/fendo.2026.1813365

**Published:** 2026-07-23

**Authors:** Biana Shtaif, Chen Menahem, Shay Henry Hornfeld, Yankel Gabet, Moshe Phillip, Galia Gat-Yablonski

**Affiliations:** 1 Gray School of Medical Sciences, Gray Faculty of Medical and Health Sciences, Tel Aviv University, Tel Aviv, Israel; 2 Felsenstein Medical Research Center, Tel Aviv University, Petah Tikva, Israel; 3 The Jesse Z and Sara Lea Shafer Institute for Endocrinology and Diabetes, National Center for Childhood Diabetes, Schneider Children’s Medical Center of Israel, Petah Tikva, Israel

**Keywords:** collagen type X, cortical bone, epiphyseal growth plate (EGP), hypertrophic chondrocytes, sex-dependent effect, SIRT1, trabecular bone

## Abstract

**Introduction:**

We investigated the role of SIRT1 in linear growth and bone structure, focusing on the response of the epiphyseal growth plate (EGP) to nutritional manipulation.

**Methods:**

A ColX-Cre driver was used to target hypertrophic chondrocytes (HZ), and generate hypertrophic specific Sirt1 knockout (CKO) mice.

**Results:**

We found that Sirt1 deletion in hypertrophic chondrocytes markedly impaired growth in males, with a 33% reduction in body weight and 10–11% shorter long bones and EGP height (P<0.05). In females, effects were milder (12% reduction in weight, 5–7% shorter long bones; P<0.05 in both) but EGP height was reduced similarly to the males. These mice were next tested in a catch-up (CU) growth model involving food restriction followed by refeeding. Under CU conditions, male CKO mice showed an unexpected exaggerated response, surpassing the weight and bone length of normally fed CKO.

**Discussion:**

We therefore conclude that targeted Sirt1 deletion in the hypertrophic zone revealed a strong sex-dependent effect on skeletal growth and bone quality. Both male and female CKO mice exhibited growth impairment under normal conditions compared to littermate control (CTL), however while male showed hyper-responsiveness to refeeding, females CKO showed only moderate alterations. This targeted approach uncovers sex-dependent role for SIRT1 in regulating growth and bone quality, moving beyond its generally understood functions in cartilage and bone development. The distinct responses to both basal conditions and nutritional stress between sexes represent a significant new finding, extending its known role beyond general cartilage and bone development.

## 1 Introduction

The marked increase in adult height observed over the past century (1), together with the strong influence of environmental factors on childhood growth, points to an important role for epigenetic mechanisms in the regulation of longitudinal skeletal growth. The epiphyseal growth plate (EGP), as the primary site of postnatal bone elongation, is highly sensitive to nutritional, hormonal, and inflammatory cues. In recent years, we and others have investigated the contribution of epigenetic regulators to the function of the EGP, studying microRNAs (2, 3), DNA methyltransferases (e.g., DNMT3) (4), histone deacetylases (HDAC1, 2, 4) (5, 6), and Sirtuin-1 (SIRT1) (2, 7, 8). SIRT1 is a member of the sirtuin family of nicotinamide adenine dinucleotide (NAD^+^)–dependent deacetylases and acts as a metabolic sensor linking cellular energy status to transcriptional regulation. Of the seven mammalian sirtuins, SIRT1 is the most extensively studied and occupies a central position in pathways controlling cell fate decisions. In the context of the EGP, SIRT1 modulates chondrocyte proliferation, hypertrophic differentiation, and survival through the deacetylation of histone and non-histone proteins (9). Although primarily nuclear, SIRT1 can translocate to the cytoplasm, further expanding its regulatory capacity Known SIRT1 targets include key regulators of cell homeostasis such as p53, E2F1, NF-κB, and FOXO transcription factors (10), as well as β-catenin (11) thereby influencing transcription, DNA repair, stress responses, and apoptosis. Consistent with these functions, SIRT1 has emerged as a critical mediator of skeletal development and EGP integrity, and its dysregulation has been linked to impaired growth and to systemic diseases with skeletal manifestations.

The primary aim of this study was to investigate the role of SIRT1 in linear growth and bone structure, with the secondary aim to examine the response of the EGP to nutritional manipulation. In our previous study, we demonstrated that deletion of *Sirt1* specifically in collagen type II-expressing cells impaired both the height and organization of the EGP (8). Notably, SIRT1 also played a critical role in regulating both cortical and trabecular bone compartments; its absence in the proliferative zone affected bone mineralization and structure (8). Because Sirt1 activity depends on NAD+ levels, which fluctuate with dietary intake and energy status, SIRT1 serves as a molecular sensor that translates nutritional cues into changes in gene expression. This establishes SIRT1 as a pivotal factor through which nutritional signals modulate growth and bone formation. Indeed, the absence of SIRT1 in the chondrocytes affected EGP both under basal conditions as well as in response to nutritional manipulation. These changes collectively resulted in less efficient catch-up (CU) growth, reflected by reductions in body weight, bone length, EGP height, and overall bone architecture (8).

Because a transient expression of Collagen type II in the brain could not be excluded (12), and to further investigate the role of SIRT1 in linear growth, the present study utilized the Cre-Collagen-X (ColX), which is selectively expressed in hypertrophic chondrocytes within the EGP (13). Given previous evidence indicating sex-specific genetic effects on bone properties (14) and epigenetic differences that are maintained independently of hormones (15, 16), we analyzed the impact of the *Sirt1* manipulation under basal conditions and in response to nutritional manipulation (food restriction followed by refeeding) separately in female and male mice. Our results show a significant difference between female and male mice, suggesting a complex and critical interplay between SIRT1 and sex hormones in growth regulation.

Linear growth of childhood usually occurs without any obstacles, but can be impaired by numerous genetic and environmental factors. The therapeutic toolbox for short stature in children is very limited; therefore, investigating the underlying molecular pathways and finding new regulatory targets is crucial for the development of novel means of treatment.

## 2 Materials and methods

### 2.1 Animals

All procedures and experiments were approved by the Institutional Animal Care and Use Committee of Tel Aviv University (committee protocol approval number 2305-133-4), before onset of the study. We used mice carrying floxed *Sirt1* alleles (B6;129-Sirt1tm1Ygu/J) generated by Dr. Yansong Gu (University of Washington School of Medicine), and provided with permission from Jackson Laboratories by Prof. Mona Dvir-Ginzberg (the Hebrew University, Israel). These *Sirt1^fl/fl^* mice were shown to reproduce the full *Sirt1*-null phenotype when crossed with a mouse ubiquitously expressing Cre (17). To selectively delete *Sirt1* in hypertrophic chondrocytes, we mated *Sirt1^fl/fl^* mice with ColX-Cre mice expressing the Cre recombinase under the control of the Collagen X promoter, which is active predominantly in hypertrophic chondrocytes (13, 18) (kindly provided by the late Prof. Itai Bab, The Hebrew University, Jerusalem, Israel). Thereafter, heterozygous *Sirt1^fl^*^/+^; ColX-Cre^+/-^ were crossed with *Sirt1^fl/fl^* mice and the resulting *Sirt1^fl^*^/^*^fl^*; ColX-Cre^+/-^ were bred with *Sirt1^fl^*^/^*^fl^* mice to generate *Sirt1^fl^*^/^*^fl^*; ColX-Cre^+/-^ (named throughout the manuscript as CKO) and their *Sirt1^fl^*^/^*^fl^*;ColX-Cre^-/-^ littermate controls (CTL).

To verify the production of the transgenic mice, DNA was extracted from mouse tails using DirectPCR lysis reagent (Viagen Biotech, Los Angeles, CA, USA) according to the manufacturer’s instructions. To detect the *Sirt1* floxed allele, we used the primers suggested by Jackson Laboratories: 0IMR7909: 5’-GGTTGACTTAGGTCTTGTCTG-3’ and 0IMR7912: 5’-CGTCCCTTGTAATGTTTCCC-3’. To detect Cre, we used the following primers: F: 5’-CCTGGAAAATGCTTCTGTCCGTTTGCC; R: 5’-GAGTTGATAGCTGGCTGGTGGCAGATG-3’. Primers for IL-2 served as an internal control for PCR reaction efficiency (IL-2 F: 5’- CTA GGC CAC AGA ATT GAA AGA TCT-3’; IL-2 R: 5’- GTA GGT GGA AAT TCT AGC ATC ATC C-3’). Mice were maintained in group cages under specific pathogen-free conditions (25 ± 1°C, humidity 50 ± 2%, 12h light/dark cycle; lights off at 18:00h) and allowed free access to chow (2018SC; Teklad Rodent Diet, Envigo, Madison, WI, USA) and water, except during the food-restriction period, as described later. The normal intake of the CTL and CKO mice was measured separately and served as reference for determining food restriction in the CU study (described in section 2.2).

### 2.2 Catch-up growth

Twenty-one-days-old mice (CKO and CTL separately) were divided into solitary cages and allowed to acclimate for 3 days, then the mice were randomly allocated to two groups: unlimited access to regular mice chow (mice fed *ad libitum*; AL group) or fed 60% of the normal daily intake of the same chow for 36 days (8, 19) to which food was administered once daily. After 36 days of 40% food restriction (with the baseline food consumption serving as a reference for each group), all restricted animals were re-fed with unlimited amounts of the same chow and followed for additional 28 days (CU group). At the end of the experiment, all mice were euthanized by CO_2_ inhalation and killed.

### 2.3 Histological staining and measurement of growth plate height

The tibiae and humeri of each animal were carefully removed, cleaned, and measured for length with a digital caliper. Tibia were used for histological measurements, and humeri were used for micro-CT analysis (section 2.7). The tibiae were fixed in 4% neutral buffered formalin, decalcified with Decalcifier-II (cat.no. 3800460E, Leica, Richmond, IL, USA), dehydrated with a graded ethanol series (70%, 95%, 100%), and stabilized by two sequential changes of chloroform for paraffin embedding. Histological studies and EGP height measurements were performed on 6μm paraffin sections using Image-Pro software (version 4.5.1.22, Media Cybernetics, Rockville, MD, USA) by two people blinded to their origin. For each bone, two sections were measured, for a total of five measurements per section. The height of the EGP was measured by drawing a straight line from the apical border of the reserve zone cells to the lower border of the mineralized cartilage.

### 2.4 Immunohistochemistry

For the immunohistochemistry study, decalcification was performed using 12.5% EDTA for 21 days. Paraffin-embedded tissue sections were de-paraffinized in xylene, rehydrated in a graded series of ethanol, and washed with PBS. Immunohistochemistry was performed at room temperature. Nonspecific background was blocked by incubation with a blocking solution consisting of 2% horse serum (VE-MP-7401-50, ImmPress Kit, Vector Laboratories, Burlingame, CA, USA) for 20 min followed by incubation with anti-Sirt1 antibody (cat # 07-131, Millipore, Temecula, CA, USA) diluted 1/200 in PBS for 2h. The sections were washed three times in PBS and incubated with anti-rabbit antibody (VE-MP-7401-50, ImmPRESS Kit, Vector Laboratories) for 30 min. PBS without antibody was used as a negative control for all sections. Aminoetyl carbazole (AEC substrate kit, cat # 00-2007, Life Technologies, Eugene, OR, USA) was added as a substrate in both cases for visualization of positive-stained cells. Counterstaining was performed with hematoxylin followed by water washout.

### 2.5 ATDC5 cell culture

The chondrogenic ATDC5 cell line (20) was cultured in Dulbecco’s Modified Eagle Medium/Ham’s-F12 (1:1 mixture, Gibco/Life Technologies, Thermo-Fisher Scientific) containing 5% heat-inactivated fetal calf serum, 1% l-glutamine and 1% penicillin/streptomycin (from IMBH, Beit HaEmek, Israel) at 37 °C in a humidified atmosphere of 5% CO_2_ in air. Cells were seeded at an initial density of 12 × 10^3^ cells/cm^2^ in a 24-multiwell plate (Corning). The next day, the cells were induced to differentiate by the addition of insulin, transferrin and sodium selenite (ITS) (Sigma-Aldrich) for 21 days to obtain cells at the hypertrophic stage of differentiation (21).

### 2.6 RNA extraction and RT-PCR

Total RNA was extracted from the patella, kidney and spleen of old mice (18–25 months) (22), using the RNeasy Mini Kit (cat. no./ID: 217004, Qiagen, Valencia, CA, USA) according to the manufacturer’s protocol. To verify Cre recombination, first-strand cDNA synthesis was performed with the PrimeScript First-Strand cDNA Synthesis Kit (Takara Bio, Mountain View, CA, USA) using 1 μg of the total RNA as a template, according to the manufacturer’s instructions. The primers for *Sirt1* knockout were from exons 3 and 5 (exon 3 forward: 5’-GACGCTGTGGCAGATTGTTA-3’; exon 5 reverse: 5’-ACACAGAGACGGCTGGAACT-3’), yielding a product size of 325bp for the normal control and 180bp for the transgene. The primers for *ColX* were: F: 5’-TGA TAT TCC TGG TGG TCC TGG CAA C; R: 5’-ATA CCC TTT CTG CTG CTA ATG TTC TTG ACC (22).

RNA extraction from ATDC5 cells - RNA was isolated using TRI reagent (Sigma Aldrich, cat. No. T9424) and chloroform, collecting the aqueous phase after centrifugation (4°C, 15 min. 14,000 rpm). RNA was precipitated for 5 min with isopropanol at room temperature, and pelleted by centrifugation (4°C, 8 min. 12,000 rpm). RNA pellets were washed in 70% ethanol, centrifuged (4°C, 5 min. 7500 rpm) and dried. RNA was dissolved in DNase/RNase-free pure water. RNA quantity and purity were determined by NanoDrop™ One Spectrophotometer (Thermo Scientific).

### 2.7 Micro-CT analysis

Micro-CT analysis was performed essentially as previously described (12). Humeri (n=6 from each group) were maintained in 4% neutral buffered formalin for 48h at room temperature and then stored in 70% ethanol. In our previous numerous studies, we found that 6 animals per groups enable us to gain the maximum information possible, under the space limitation (2, 8, 23) and the differences between the groups were statistically significant.

The entire right humerus was scanned using a micro-CT system (μCT50; Scanco Medical AG, Switzerland). When the right sided bone was not intact, the left one was used instead. Scans were acquired at 90 kVp energy, 88 μA intensity, and 1100 ms integration time, generating images with an isotropic nominal resolution of 10 μm. To ensure accurate mineral density measurements, we checked the x-ray tube signal every 1–2 weeks using the manufacturer-provided calibration phantom; recalibration was performed when the signal fluctuated by more than 2.5%. A three-dimensional Gaussian filter was used to attenuate the background noise in the volumes (σ= 0.8; support=1). The scans were segmented using a global thresholding procedure (with a threshold of 268 mgHA/cm^3^ for trabecular bone and 535 mgHA/cm^3^ for cortical bone. Morphometric parameters were determined by a direct three-dimensional approach in three different pre-selected regions of interest using customized software developed on the proprietary Image Processing Language, version 7.1 (Scanco Medical).

In the whole bone, we measured humerus length, bone volume fraction (bone volume/total volume, BV/TV [%]) and volumetric bone mineral density (vBMD). In the cortical bone, we used a diaphyseal segment measuring 1 mm in height, starting at the 60^th^ percentile of the humerus length. Cortical measurements included Volumetric Bone Mineral Density (vBMD), total area (Tt.Ar, mm^2^), cortical area (Ct.Ar, mm^2^), cortical area fraction (Ct.Ar/Tt.Ar, %), cortical thickness (Ct.Th, mm) and tissue mineral density (TMD). The trabecular bone compartment consisted of a 2-mm height volume of interest in the secondary spongiosa in the proximal metaphysis of the humeri, separated manually from the cortical bone by tracing the endosteal surface on the axial 2D tomographic slices, and starting proximally at the distal limit of the primary spongiosa. Measurements included trabecular BV/TV, number (Tb.N, mm^−1^), thickness (Tb.Th, mm), and separation (Tb.Sp, mm). The proximal metaphysis of the humerus was then divided into two 1-mm halves along the longitudinal axis, named proximal and distal subregions (the proximal and distal bone compartments are from the proximal humerus).

### 2.8 Statistical analysis

Comparisons between the groups for animals’ weight were analyzed using two-way repeated measure analysis of variance (RM-ANOVA) or Two-way Mixed model analysis. Since the result showed significant interactions, we used T-Tests for each body weight measurement. For each time point, an F-test for equality of variances was conducted to assess homogeneity of variance. When the variances were not significantly different (p ≥.05), a standard independent samples T-test assuming equal variances was used. When the variances differed significantly (p <.05), Welch’s T-test (unequal variances) was applied. T-Test analysis was used between each pair of experimental groups, for linear growth parameters and micro-CT analysis, as only specific comparisons were of biological significance. Data are presented as mean ± SD. Statistical significance was set at p < 0.05. All analyses were performed using GraphPad Prism (version 9).

## 3 Results

### 3.1 Verification of the genetic manipulation

**Figure 1:**
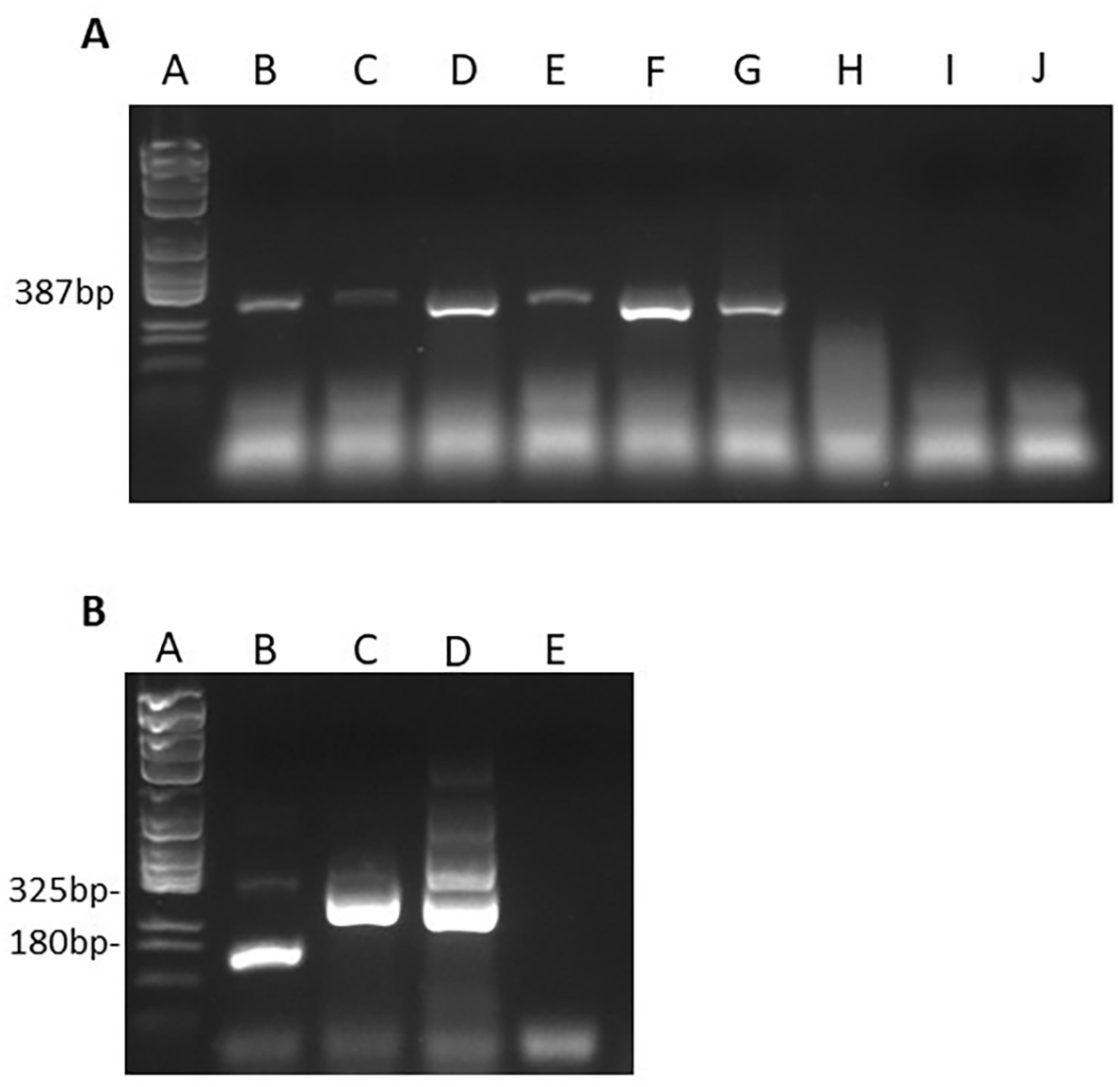
Reverse transcriptase PCR of Col X and Sirt1 exon 4 deletion in RNA extracted from patellas of old mice. **(A)** Col X expression. A-marker; B-Patella extracted from CKO mice at age 18-21m; C-Patella extracted from CKO mice age 22-25m; D-Patella extracted from C57 black mice (WT) age 18m; E- Patella extracted from ColII-CKO mice age 18m; F- Patella extracted from ColII-CTL mice age 18m (8); G-ATDC5-21d; H-Kidney of CKO mice; I-Spleen of CKO mice; J-water; **(B)** DNA recombination of collagen X-specific Cre-Sirt1 was confirmed by the presence of the recombined allele in cartilage from the CKO mice but not in the CTL or ATDC5 cells. A-marker; B-Patella extracted from CKO mice age 22-25m; C-Patella from C57 black-WT age 18m; D-ATDC5-21d; E-water.

ColX-Cre-recombinase removes exon 4 of the floxed *Sirt1* gene (encoding the catalytic domain of the protein) (17, 24) specifically in hypertrophic chondrocytes. Immunohistochemistry in the EGP of growing mice showed a significant reduction in the level of Sirt1 protein (**Supplementary Figure 1**), with no significant change in the structure of the EGP (**Supplementary Figure 2**). In addition, as the hypertrophic zone (HZ) of the mice was very narrow, we extracted the RNA from patella of old mice (22), with differentiated ATDC5 cells serving as control. As shown by van der Kraan et al, ColX expression is restricted to the HZ of the EGP; however, in old mice (18–25 months) ColX is also expressed in the patella, whereas it is absent from the patellae of younger animals. Indeed, the presence of ColX expression in this tissue enabled us to verify the efficient knockout of SIRT1 in ColX-expressing cells. To ensure Cre recombination, we verified, using RT-PCR, that ColX was expressed in all the patellas and in ATDC5 cells but not in the kidney and spleen, as expected (**Figure 1A**). DNA recombination of ColX-specific Cre-*Sirt1* was confirmed by the presence of the recombined allele in cartilage from the CKO mice but not in the CTL or ATDC5 cells (**Figure 1B**); the loss of exon 4 was confirmed by sequencing.

Further analysis is presented separately for male and females, due to the significant differences between these groups.

### 3.2 Males

#### 3.2.1 Effect on body weight

**Figure 2:**
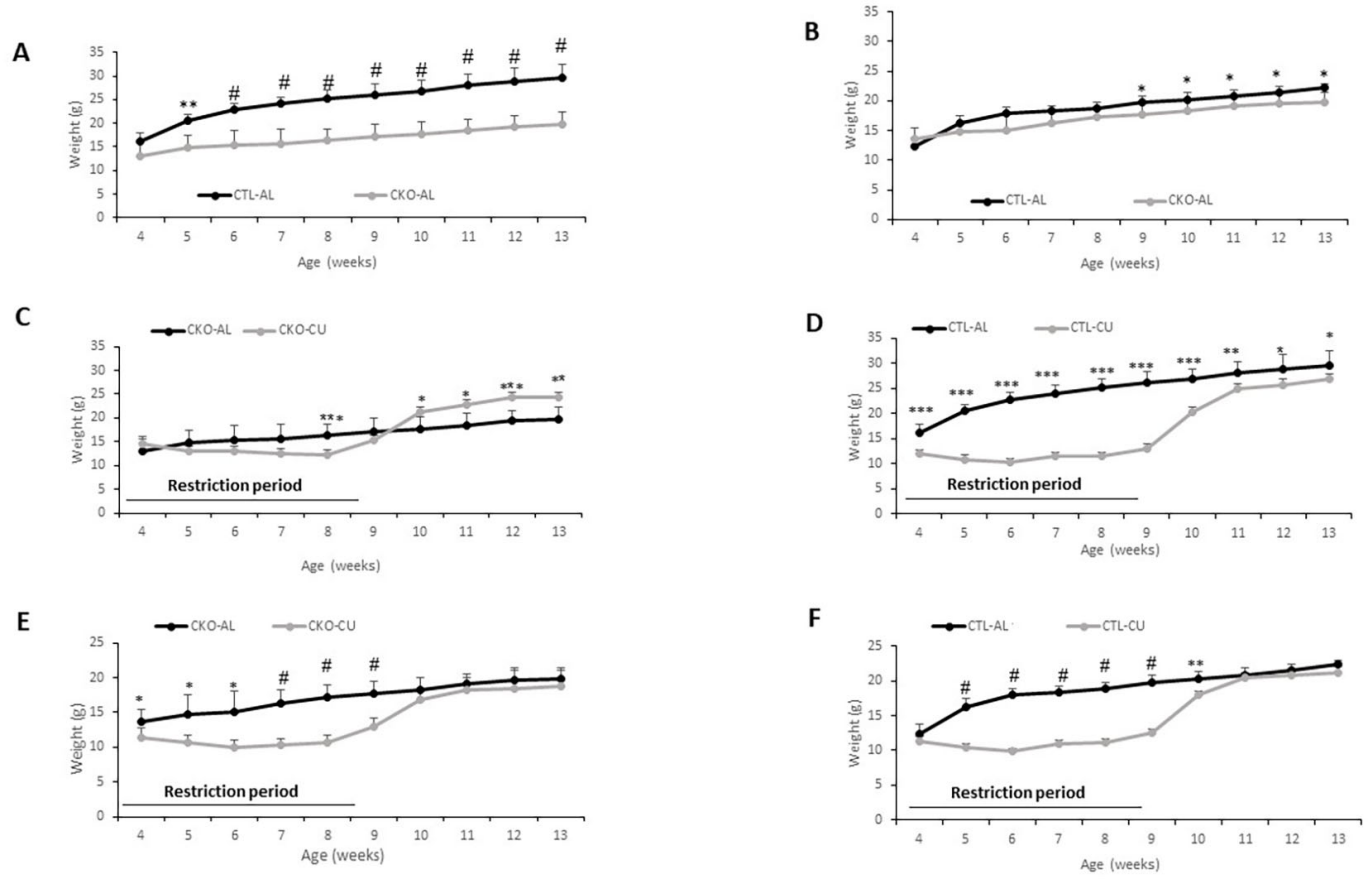
Follow-up of body weight. **(A, B)** under AL feeding conditions. A-male mice; B-female mice; **(C–F)** Weight changes in AL and CU. C-CKO male mice; D-CTL male mice; E-CKO female mice; F-CTL female mice; *p<0.05; **p<0.005; ***p<0.001; ^#^p<0.0001.

The animals showed no difference in size and weight at birth. However, with increasing age there was a significant difference in the change over time between the CKO and CTL male mice, with a greater increase in weight gain in the CTL group (**Figure 2A**; n≥12). Food consumption was measured at the age of 24-40d in order to calculate the amount of food provided during the food restriction period. Interestingly, there was a significantly lower food intake in the CKO males (2.43 ± 0.27gr/day) than in CTL counterparts (2.84 ± 0.11gr/day; p<0.05).

#### 3.2.2 Effect of CU growth

At the end of the refeeding period (age of 88 days), the average weight was significantly higher in CTL mice fed AL compared to CKO-AL, and also in CTL mice that were exposed to food restriction followed by refeeding (CTL-CU) compared to CKO-CU. Interestingly, the weight of the animals of the CKO-CU group exceeded that of their controls (CKO-AL), a phenomenon we have never seen before (**Figures 2C, D**) (8, 21, 23). Similar findings were observed for the length of the tibia and the humerus, namely, the length was greater in the CTL-AL animals compared to CKO-AL; in the CTL animals the bones were shorter in the CU compared to AL, while it was increased in CKO-CU to reach a level that was higher than CKO-AL (**Table 1**), but still did not reach the weight or length of the CTL-CU. However, while in both CKO-CU and CTL-CU there was an increase in EGP height compared to their AL controls, similar to our previous data, the EGP height did not differ between CKO and CTL (**Table 1**), probably due to their age. We did not observe any gross morphological differences in EGP structure between CTL and CKO mice in the histological sections at this age also (data not shown).

**Table 1:** Comparison of anthropometric and bone parameters between CKO and CTL male mice under two nutritional conditions (AL and CU).

	CKO		CTL		P1	P2	P3	P4
	AL	CU	AL	CU				
Anthropometric Parameters								
Body Weight (gr)	19.65 ± 2.63	24.44 ± 2.20	29.50 ± 2.92	26.79 ± 1.08	0.007	0.022	<0.0001	0.0063
Tibia Length (mm)	17.14 ± 0.23	17.66 ± 0.16	18.81 ± 0.45	17.92 ± 0.12	0.022	<0.0001	<0.00001	0.0011
Humerus Length (mm)	11.35 ± 0.30	11.78 ± 0.20	12.80 ± 0.31	12.03 ± 0.14	0.019	<0.0001	<0.00001	0.02
EGP (mm)	0.094 ± 0.015	0.118 ± 0.01	0.106 ± 0.009	0.117 ± 0.008	0.01	0.059	0.138	0.866
Cortical Bone Parameters								
vBMD	816.22 ± 36.26	825.55 ± 48.28	823.47 ± 25.72	803.84 ± 31.1	0.722	0.261	0.698	0.416
Tt.Ar (mm^2)	0.68 ± 0.04	0.71 ± 0.05	0.86 ± 0.06	0.76 ± 0.03	0.363	0.003	0.0001	0.097
Ct.Ar (mm^2)	0.44 ± 0.03	0.47 ± 0.03	0.55 ± 0.02	0.50 ± 0.02	0.162	0.003	0.00002	0.057
Ct.Ar./Tt.Ar (%)	55.27 ± 2.66	66.34 ± 3.48	64.72 ± 2.36	66.10 ± 1.55	0.576	0.260	0.715	0.891
Ct.Th (mm)	0.19 ± 0.01	0.20 ± 0.01	0.21 ± 0.01	0.20 ± 0.01	0.163	0.815	0.009	0.388
TMD	1296.33 ± 10.65	1283.39 ± 13.15	1311.30 ± 15.49	1249.78 ± 27.60	0.104	0.001	0.073	0.03
Trabecular Bone Parameters								
BV/TV (%)	8.59 ± 2.48	10.47 ± 2.61	11.28 ± 2.18	12.32 ± 2.62	0.254	0.473	0.074	0.272
Tb.Th (mm)	43.58 ± 2.66	45.40 ± 6.40	45.35 ± 2.34	48.47 ± 1.54	0.539	0.021	0.250	0.349
Tb.N (mm-1)	3.92 ± 0.85	3.85 ± 0.53	4.68 ± 0.47	4.14 ± 1.09	0.871	0.297	0.086	0.574
Tb.Sp (mm)	263.82 ± 70.93	264.96 ± 38.21	211.32 ± 25.97	260.95 ± 88.74	0.975	0.218	0.119	0.923
Conn.D	81.24 ± 43.54	138.41 ± 28.23	121.65 ± 30.43	155.54 ± 57.02	0.033	0.228	0.092	0.537
SMI	2.70 ± 0.17	2.65 ± 0.22	2.80 ± 0.22	2.67 ± 0.17	0.643	0.289	0.421	0.861
TMD	798.0 ± 26.29	772.57 ± 20.24	785.49 ± 5.86	758.47 ± 31.62	0.11	0.067	0.282	0.394

Results are presented as mean ± SD. T-Test analysis was used between each pair of experimental groups, as only specific comparisons were of biological significance.

CU-Mice were subjected to 36 days of 40% food restriction followed for unlimited refeeding for additional 28 days.

CKO- collagen type X-specific Sirt1 knockout mice; CTL- Sirt1 flox/flox control mice without the Cre transgene; AL-fed ad libitum; CU-re-fed (catch-up growth);EGP-epiphyseal growth plate; vBMD-volumetric bone mineral density; BV/TV-bone volume fraction; Tt.Ar-total area; Ct.Ar-cortical area; Ct.Th-cortical thickness; TMD-tissue mineral density; Tb.Th-trabecular thickness; Tb.N-trabecular number; Tb.Sp-trabecular separation; Conn.D-connectivity density; SMI-structure model index.

P1-T-test comparison between CKO-AL and CKO-CU; P2- T-test comparison between CTL-AL and CTL-CU; P3-T-test comparison between CKO-AL and CTL-AL; P4-T-test comparison between CKO-CU and CTL-CU.

#### 3.2.3 Micro CT analysis

Results of the micro-CT analysis performed on the humeri from the 4 male groups are presented also in **Table 1**. There were no significant differences in cortical vBMD, but total cross-sectional area (Tt.Ar), cortical bone area (Ct.Ar) and cortical thickness (Ct.Th) were all significantly higher in the CTL group compared to CKO. Nutritional manipulation significantly reduced cortical parameters in the CTL mice, while it had no effect on CKO mice. Most trabecular bone parameters were not significantly affected; however, Tb.Th was higher in CTL-CU compared to CTL-AL, with no parallel effect in the CKO groups. In contrast, the connectivity, which tended to be lower in the CKO compared to CTL, was significantly increased in the CKO mice after refeeding, while only a marginal effect was observed in the CTL mice. It seems that the CKO mice were significantly less sensitive to the nutritional changes compared to the CTL mice in cortical bone parameters (**Table 1**), similar to our previous observation regarding the Col II-Cre SIRT1 line (8), although most of the bone parameters were similarly corrected after refeeding (**Table 1**). Overall, the effect of food restriction and CU was less pronounced in the CKO male mice in terms of bone mineralization and structure in both the cortical and trabecular bone compartments.

##### 3.2.3.1 Analysis of proximal and distal subregions

**Figure 3:**
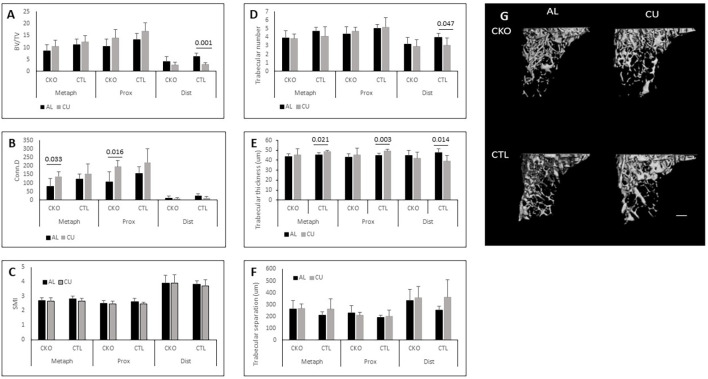
Effect of nutritional manipulation on humeri trabecular bone parameters in male mice (proximal and distal bone compartments). **(A)** BV/TV; **(B)** Conn.D-connectivity density; **(C)** SMI-structure model index; **(D)** trabecular number; **(E)** trabecular thickness; **(F)** trabecular separation; **(G)** Representative 3D model of micro-CT findings.

In growing mice, EGP moves away from the diaphysis and remodeling replaces the calcified cartilage by woven and trabecular bone. At the end of our study, it is likely that the trabecular bone at the distal part of the metaphysis replaced the EGP at the time of food restriction while the proximal part is formed during the CU phase in the CU groups. When analyzed separately the distal and proximal subregions of the metaphysis (both from the proximal humerus) (**Figure 3**), CTL male mice exhibited a reduction in BV/TV in the distal region, accompanied by decreased trabecular number and thickness. This indicates that food restriction impaired trabecular bone micro-architecture in the region that was close to the EPG at that time, whereas upon refeeding (CU), we observed an increased Tb.Th (and a trend for higher BV/TV and Tb.N) in the proximal region. In contrast, CKO male mice displayed only minor changes in trabecular bone parameters across both regions, suggesting less sensitivity to nutritional manipulations in the absence of SIRT1. The only notable alteration was an increased connectivity density in the proximal section after refeeding (CU), which may be indicative of altered remodeling of the primary spongiosa into trabecular bone and could be associated with a relatively higher proportion of woven bone in the CKO mice.

### 3.3 Females

#### 3.3.1 Effect on body weight

The female pups showed no difference in size and weight at birth. With increasing age there was a slight difference in the change over time between the CKO and CTL female mice, with tendency to greater weight gain in the CTL group, that reached statistical significance from day 9 onwards (**Figure 2B**; **Table 2**; n≥12). There was no significant difference in food consumption between CKO and CTL female mice (average food consumption was 2.28 ± 0.24 gr/day for both), which was lower compared to the males.

**Table 2:** Comparison of anthropometric and bone parameters between CKO and CTL female mice under two nutritional conditions (AL and CU).

	CKO		CTL		P1	P2	P3	P4
	AL	CU	AL	CU				
Anthropometric Parameters								
Body Weight (gr)	19.76 ± 1.73	18.74 ± 2.4	22.33 ± 0.63	21.22 ± 0.84	0.39	0.09	0.027	0.018
Tibia Length (mm)	17.2 ± 0.28	16.86 ± 0.47	18.13 ± 0.33	17.58 ± 0.23	0.122	0.006	0.00014	0.0015
Humerus Length (mm)	11.45 ± 0.22	11.18 ± 0.30	12.14 ± 0.26	11.58 ± 0.18	0.085	0.001	0.0002	0.017
EGP (mm)	0.084 ± 0.01	0.106 ± 0.01	0.098 ± 0.005	0.11 ± 0.01	0.0026	0.037	0.019	0.461
Cortical Bone Parameters								
vBMD	836.39 ± 40.78	823.35 ± 41.94	815.99 ± 26.12	785.43 ± 20.83	0.597	0.064	0.326	0.09
Tt.Ar (mm^2)	0.68 ± 0.05	0.62 ± 0.03	0.74 ± 0.04	0.68 ± 0.04	0.028	0.042	0.030	0.027
Ct.Ar (mm^2)	0.47 ± 0.03	0.42 ± 0.02	0.51 ± 0.03	0.45 ± 0.02	0.006	0.003	0.022	0.01
Ct.Ar./Tt.Ar (%)	68.84 ± 2.60	67.52 ± 2.74	68.69 ± 2.55	66.46 ± 1.8	0.412	0.137	0.921	0.465
Ct.Th (mm)	0.20 ± 0.01	0.19 ± 0.01	0.21 ± 0.02	0.19 ± 0.00	0.064	0.024	0.20	0.261
TMD	1252.41 ± 28.13	1261.07 ± 13.47	1219.92 ± 27.28	1214.67 ± 12.40	0.512	0.702	0.070	0.0002
Trabecular Bone Parameters								
BV/TV (%)	6.89 ± 1.17	8.51 ± 2.69	10.39 ± 1.89	9.95 ± 4.10	0.207	0.816	0.003	0.524
Tb.Th (mm)	43.40 ± 2.38	44.18 ± 2.50	51.15 ± 3.39	47.92 ± 3.75	0.590	0.167	0.01	0.100
Tb.N (mm-1)	3.35 ± 0.48	3.51 ± 0.55	3.67 ± 0.74	3.35 ± 0.86	0.598	0.533	0.398	0.735
Tb.Sp (mm)	302.83 ± 47.94	290.17 ± 45.18	284.12 ± 65.79	320.66 ± 90.72	0.648	0.458	0.586	0.521
Conn.D	49.55 ± 16.3	92.52 ± 46.82	63.15 ± 17.13	103.31 ± 61.96	0.060	0.159	0.189	0.758
SMI	2.86 ± 0.16	2.73 ± 0.27	2.64 ± 0.10	2.67 ± 0.23	0.329	0.812	0.018	0.701
TMD	791.08 ± 21.17	764.71 ± 23.09	794.98 ± 18.25	765.42 ± 21.94	0.066	0.037	0.74	0.959

Results are presented as mean ± SD. T-Test analysis was used between each pair of experimental groups, as only specific comparisons were of biological significance.

CU-Mice were subjected to 36 days of 40% food restriction followed for unlimited refeeding for additional 28 days.

CKO- collagen type X-specific Sirt1 knockout mice; CTL- Sirt1 flox/flox control mice without the Cre transgene; AL-fed ad libitum; CU-re-fed (catch-up growth);EGP-epiphyseal growth plate; vBMD-volumetric bone mineral density; BV/TV-bone volume fraction; Tt.Ar-total area; Ct.Ar-cortical area; Ct.Th-cortical thickness; TMD-tissue mineral density; Tb.Th-trabecular thickness; Tb.N-trabecular number; Tb.Sp-trabecular separation; Conn.D-connectivity density; SMI-structure model index

P1-T-test comparison between CKO-AL and CKO-CU; P2- T-test comparison between CTL-AL and CTL-CU; P3-T-test comparison between CKO-AL and CTL-AL; P4-T-test comparison between CKO-CU and CTL-CU.

#### 3.3.2 Effect of CU growth

At the end of the refeeding period (age of 88 days), average weight was significantly higher in CTL female mice fed AL compared to CKO-AL and also the weight of the CTL mice that were exposed to food restriction followed by refeeding (CTL-CU) was greater than CKO-CU (**Table 2**). However, in contrast to the results of the males, in each line, the weight of the CU group was similar to that of the respective AL group (**Figures 2E, F**). The long bones (humerus and tibia) were significantly shorter in the female CKO fed AL compared to the CTL-AL (**Table 2**). While in the CTL, food supplementation failed to fully restore long bone length, which remained reduced relative to AL-fed controls, in the CKO animals refeeding led to full recovery, although the bones were still shorter compared to CTL-CU. EGP height from CKO female mice was significantly shorter (p=0.019) compared to the CTL mice and in both cases the EGP height in the CU group was greater compared to the AL. As in the male groups, we did not observe any morphological changes between CTL and CKO female mice at the histological level.

#### 3.3.3 Micro CT analysis

Results of the micro-CT analysis performed on the humeri from the 4 female groups are presented in **Table 2**. There were no significant differences in cortical vBMD, however Tt.Ar and Ct.Ar were significantly higher in the CTL group (CTL-AL vs CKO-AL). Nutritional manipulation significantly reduced cortical parameters in the female of both CTL and CKO mice, and the values were significant lower in the CKO mice compared to the CTL in both the AL and refed conditions. Trabecular BV/TV and Tb.Th were significantly lower in the CKO female mice compared to their controls. Most trabecular bone parameters were not significantly altered by the nutritional manipulation; however, a closer examination of the values suggests that the response pattern in CKO mice was opposite to that observed in CTL animals.

##### 3.3.3.1 Analysis of proximal and distal subregions

**Figure 4:**
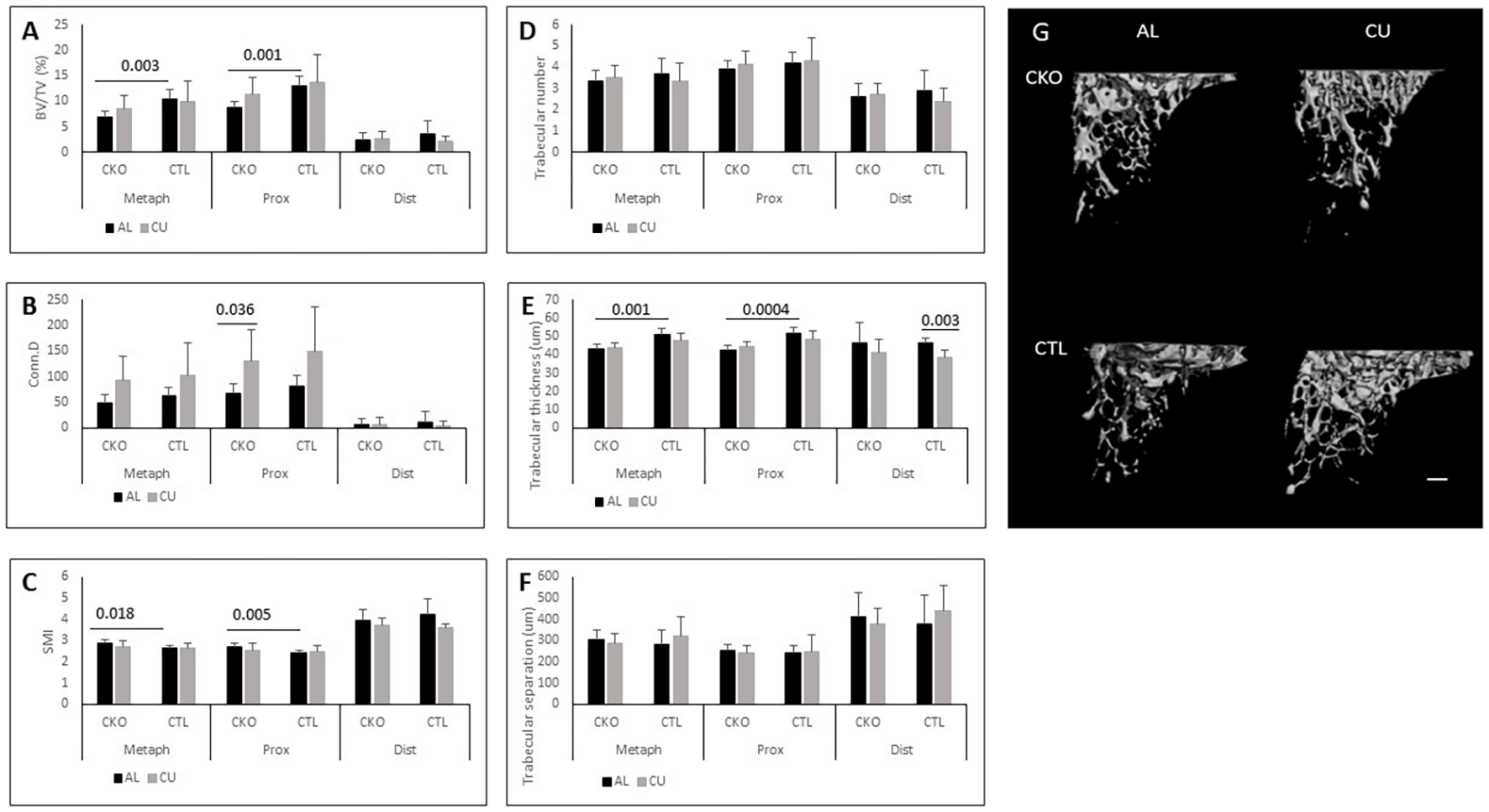
Effect of nutritional manipulation on humeri trabecular bone parameters in female mice (proximal and distal bone compartments). **(A)** BV/TV; **(B)** Conn.D-connectivity density; **(C)** SMI-structure model index; **(D)** trabecular number; **(E)** trabecular thickness; **(F)** trabecular separation; **(G)** Representative 3D model of micro-CT findings.

When the metaphysis of the humeri bone in female mice was divided into distal and proximal bone fractions, we found that the two lines differed in their response to nutritional manipulation: connectivity was increased in the proximal region in CKO female mice in the CU group, while Tb. Th was reduced in CTL mice in the distal region (**Figure 4**).

### 3.4 Effect of genotype and sex

**Figure 5:**
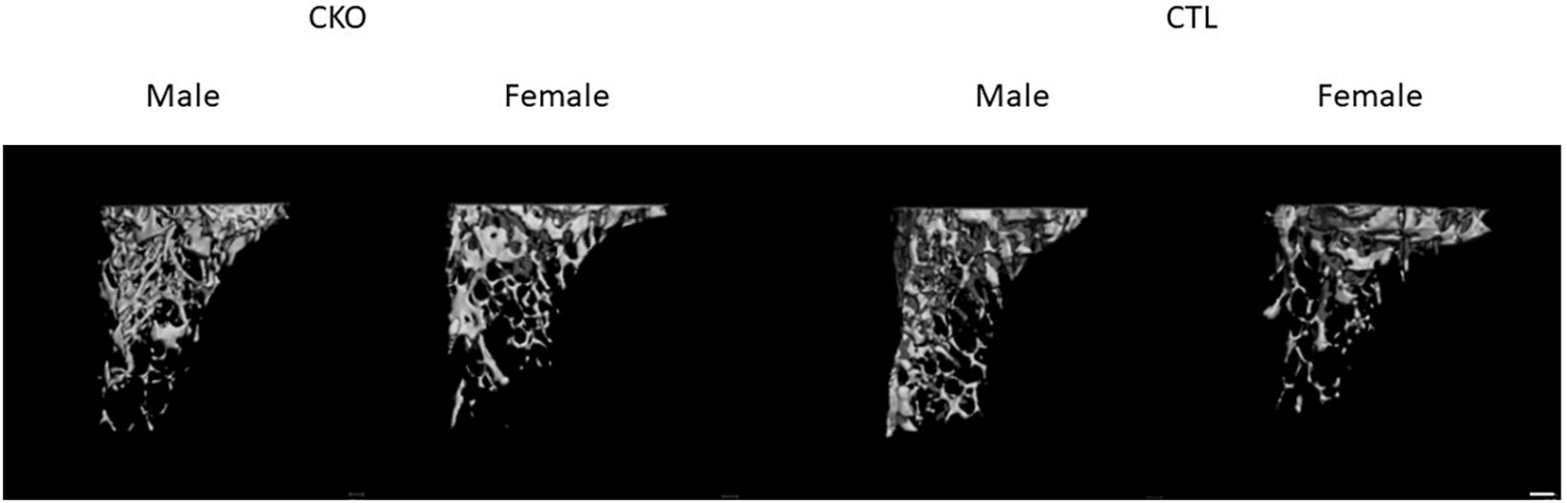
Representative 3D model of micro-CT findings in trabecular bone of male and female mice under AL feeding conditions.

Comparison between male and female mice within each genotype revealed significant sex-related differences in body weight, tibial and humeral length in the CTL mice but not in the CKO. Similarly, bone cortical and trabecular parameters were higher in the male CTL compared to female, while in CKO mice the differences were only in cortical area fraction p=0.04, cortical thickness p=0.046, and tissue mineral density p=0.005 (**Table 3**; **Figure 5**).

**Table 3:** Comparison between male and female mice.

	CKO		CTL		P1	P2
	Male	Female	Male	Female		
Anthropometric Parameters						
Body Weight (gr)	19.65 ± 2.63	19.76 ± 1.73	29.50 ± 2.92	22.33 ± 0.63	0.937	0.0006
Tibia Length (mm)	17.14 ± 0.23	17.2 ± 0.28	18.81 ± 0.45	18.13 ± 0.33	0.781	0.0054
Humerus Length (mm)	11.35 ± 0.30	11.45 ± 0.22	12.80 ± 0.31	12.14 ± 0.26	0.528	0.0009
EGP (mm)	0.094 ± 0.015	0.084 ± 0.01	0.106 ± 0.009	0.098 ± 0.005	0.183	0.081
Cortical Bone Parameters						
vBMD	816.22 ± 36.26	836.39 ± 40.78	823.47 ± 25.72	815.99 ± 26.12	0.389	0.628
Tt.Ar (mm^2)	0.68 ± 0.04	0.68 ± 0.05	0.86 ± 0.06	0.74 ± 0.04	0.993	0.002
Ct.Ar (mm^2)	0.44 ± 0.03	0.47 ± 0.03	0.55 ± 0.02	0.51 ± 0.03	0.172	0.012
Ct.Ar./Tt.Ar (%)	55.27 ± 2.66	68.84 ± 2.60	64.72 ± 2.36	68.69 ± 2.55	0.04	0.019
Ct.Th (mm)	0.19 ± 0.01	0.20 ± 0.01	0.21 ± 0.01	0.21 ± 0.02	0.046	0.254
TMD	1296.33 ± 10.65	1252.41 ± 28.13	1311.30 ± 15.49	1219.92 ± 27.28	0.005	<0.0001
Trabecular Bone Parameters						
BV/TV (%)	8.59 ± 2.48	6.89 ± 1.17	11.28 ± 2.18	10.39 ± 1.89	0.161	0.467
Tb.Th (mm)	43.58 ± 2.66	43.40 ± 2.38	45.35 ± 2.34	51.15 ± 3.39	0.902	0.0025
Tb.N (mm-1)	3.92 ± 0.85	3.35 ± 0.48	4.68 ± 0.47	3.67 ± 0.74	0.182	0.0098
Tb.Sp (mm)	26.82 ± 70.93	302.83 ± 47.94	211.32 ± 25.97	284.12 ± 65.79	0.291	0.014
Conn.D	81.24 ± 43.54	49.55 ± 16.3	121.65 ± 30.43	63.15 ± 17.13	0.126	0.0021
SMI	2.70 ± 0.17	2.86 ± 0.16	2.80 ± 0.22	2.64 ± 0.10	0.136	0.137
TMD	798.0 ± 26.29	791.08 ± 21.17	785.49 ± 5.86	794.98 ± 18.25	0.161	0.253

Values are presented as mean ± SD.

Mice were refed after 36 days of 40% food restriction and followed for 28 days.

CKO- collagen type X-specific Sirt1 knockout mice; CTL- Sirt1 flox/flox control mice without the Cre transgene; AL-fed ad libitum;; CU-re-fed (catch-up growth).;EGP-epiphyseal growth plate; vBMD-volumetric bone mineral density; BV/TV-bone volume fraction; Tt.Ar-total area; Ct.Ar-cortical area; Ct.Th-cortical thickness; TMD-tissue mineral density; Tb.Th-trabecular thickness; Tb.N-trabecular number; Tb.Sp-trabecular separation; Conn.D-connectivity density; SMI-structure model index.

**P1**- *p*-values from *t*-test comparing CKO male and female; **P2**- *p*-values from *t*-test comparing CTL male and female.

T-Test analysis was used to compare each pair of experimental groups, as only specific comparisons were biologically significant. **P1-** *p*-values from *t*-test comparing CKO male and female; **P2**- *p*-values from *t*-test comparing CTL male and female.

## 4 Discussion

There are several important findings in this study: the first is that Sirt1 deletion in hypertrophic chondrocytes markedly impairs growth. In addition, we found differential effects of SIRT1 KO in male versus female on both EGP and bone microarchitecture. This is a fascinating aspect of skeletal biology, and it may stem from sex-specific genetic effects on bone properties (14, 25), epigenetic differences (15) as well as the distinct hormonal milieus and signaling pathways present in each sex. Deletion of Sirt1 in the HZ profoundly altered all growth parameters in the affected male mice with a reduction of 33% in weight and 10-11% in both long bones’ length and EGP height, while in females the differences were more subtle (12% in weight, 5-7% in long bones length) with a similar effect on EGP height. This suggests that while SIRT1 is essential for optimal endochondral ossification in both sexes, the male skeleton is significantly more vulnerable to its loss during periods of rapid postnatal growth. Furthermore, under CU conditions, male CKO mice showed an unexpected exaggerated CU response, surpassing the weight and bone length of normally fed CKO, this implies that in males, SIRT1 might normally act as a regulator of the recovery phase, and its absence removes a specific constraint on the EGP’s anabolic potential. Our results clearly show that SIRT1’s role in the EGP cannot be understood without considering the specific hormonal context of each sex.

Sirtuin 1 (SIRT1) is a multifunctional protein involved in numerous cellular processes. Mice lacking Sirt1, or carrying a point mutation in the gene, exhibit markedly reduced body size (17), implicating Sirt1 in EGP chondrogenesis and its contribution to longitudinal bone growth. In the EGP, SIRT1 enhances the expression of cartilage-specific genes, including those encoding components of the extracellular matrix (26), support chondrocyte proliferation and differentiation, and promote chondrocyte survival (27), thereby contributing to proper development.

In our previous investigations of the regulatory mechanisms underlying CU growth, we observed that miR-140 and miR-22 were downregulated in the EGP of food-restricted rats. Both microRNAs target Sirt1, whose protein levels showed a corresponding twofold increase (8, 21). In our previous study, we reported that transgenic Cre Col II- Sirt1 CKO mice, in which from the proliferative zone, had shorter bones, a disorganized EGP, reduced bone mineralization, increased body weight, and diminished responsiveness to nutritional manipulation, resulting in less efficient CU growth (8). In the present study, we observed that Cre ColX-*Sirt1* knockout (CKO) mice exhibited also significantly reduced bone length, along with lower body weight and decreased food intake, compared to their littermates. CKO mice were again, significantly less sensitive to food restriction with regards to bone quality. Furthermore, to our surprise, the effect of nutritional manipulation on the CKO male mice was to increase body weight and linear growth, a phenomenon that may be associated with the removal of the control of SIRT1.

To better define the role of SIRT1 in skeletal development and avoid potential confounding effects arising from embryonic collagen II expression in non-skeletal tissues (28, 29), we used a ColX-Cre model to delete Sirt1 specifically in hypertrophic chondrocytes. However, as in our previous model of Cre col II-Sirt1 KO, in which we showed reduced activity, heightened anxiety, and slower learning (12), there was also a subtle effect on animal behavior in the current model. CKO mice exhibited a significantly less anxious behavioral profile or increased curiosity (data not shown). This further suggest a central connection between bone and brain, that is associated with Sirt.

One potential mediator linking the observed alterations in both bone and behavior is undercarboxylated Osteocalcin (ucOC), which serves as a marker of bone turnover and has also been implicated in the bone–brain axis. ucOC has been shown to cross the blood–brain barrier and influence cognitive function by promoting learning and memory, enhancing neurotransmitter synthesis, and reducing anxiety- and depression-like behaviors (30). Therefore, we measured circulating ucOC levels to determine whether changes in bone dynamics or bone-derived endocrine signaling could contribute to the observed phenotype. However, endpoint measurements revealed no significant differences in ucOC levels between Cre-Col II-SIRT1 CKO mice (12), Cre-ColX-SIRT1 CKO mice (data not shown), and their respective controls, suggesting that neither altered dynamic bone formation nor ucOC-mediated bone-to-brain signaling is likely to explain the observed effects.

Both genetic and hormonal factors contribute to the observed sexual dimorphism in bone density and structure in mice. Most studies, but not all, show that the cortical and trabecular bone parameters in the long bones and vertebrae exhibit sexual dimorphism (14, 16, 25), with males generally having larger and potentially stronger bones than females. Indeed, in our study, CTL male mice had greater body weight, stronger and longer bones, as well as higher trabecular bone density than their female counterparts. There are many examples of genes playing sex-specific skeletal roles in mice [i.e. Lef1 31 (16); Krox20 (15)]. It was also reported that sexual dimorphism may be associated with sex-specific epigenetic changes that suppress genes involved in skeletal homeostasis (15), a mechanism that could be attributed to Sirt1.

The differential effects of SIRT1 knockout on CU growth in males and females is a new example of sex-by-gene interaction. The sexual dimorphism may result from interactions between SIRT1 and sex hormones in the EGP. Previous studies have shown the importance of estrogen receptor (ER) in the EGP for proper growth and growth cessation (30, 31). Given our prior demonstrations of aromatase activity in EGP chondrocytes (32), and its response to leptin (33), further support the connection between nutrition, sex hormones and growth. Within the EGP, locally produced estrogens signal through ER to regulate chondrocyte proliferation, differentiation, and hypertrophy, all processes essential for endochondral ossification and longitudinal bone growth (31). These effects may be mediated, at least in part, through modulation of SIRT1 expression and activity, as accumulating evidence indicates a complex and multifaceted interplay between SIRT1 and estrogen in the regulation of diverse physiological processes (34). Estrogen has been reported to upregulate SIRT1 expression in the liver (35), and an interaction between SIRT1 and ERα has been demonstrated in breast cancer cells (36). Within the EGP, these interactions may influence the transcription of genes critical for chondrocyte proliferation and differentiation. Supporting this notion, studies in articular cartilage have shown that estrogen protects chondrocytes by enhancing mitophagy through a SIRT1-dependent signaling pathway (37), suggesting a mechanism by which estrogen, via SIRT1, preserves cellular integrity and delays premature chondrocyte aging. Even though estrogen is important for both sexes, the way EGP chondrocytes respond to estrogen can be sex specific. For example, some studies indicate that estradiol effects on intracellular calcium signaling in chondrocytes are more pronounced in cells from female than from male rats (38). This suggests that the downstream signaling pathways activated by ER might differ between the sexes. In females, SIRT1 may be more directly and robustly involved in the estrogen-mediated signaling that drives EGP maturation and fusion, as females were more resilient to the nutritional challenge, with their weight remaining similar between the AL and CU groups. Female mice show milder reductions in bone length than males resulting from SIRT1 loss, but bone mass and architecture deficits persist in both sexes. This suggests a possible bone-sparing effect of estrogen or other female factors, even in the absence of SIRT1 expression in EGPs. Estrogen generally promotes skeletal accrual and protects bone quality during energy restriction and adolescence. The less severe phenotype in CKO females versus males, underlines estrogen’s compensatory effect that partially mitigates the loss of SIRT1 in the HZ. It should be noted, however, that SIRT1 was removed only in the last part of the EGP, including the HZ and the calcifying region. Consequently, the continued presence of SIRT1 in the resting and proliferative chondrocytes may have been sufficient to sustain estrogen/SIRT1–dependent signaling and preserve EGP function. Estrogen may compensate for the loss of SIRT1 through several integrated physiological and molecular mechanisms, resulting in the significantly milder growth impairment observed in female mice.

In males, the residual mechanisms (such as the conversion of testosterone to estrogen via aromatase) appear insufficient to provide the same level of protection when SIRT1 is absent in the hypertrophic cells. SIRT1 in the male HZ appears to act as a molecular sensor that translates nutritional cues into regulated growth; its absence removes constraints on recovery but leaves the skeleton with a persistent structural deficit; the anabolic response to refeeding is no longer constrained or properly modulated, allowing for a volatile, exaggerated surge in growth parameters once nutrition is restored. In the male HZ, the loss of this sensor appears to disrupt the EGP’s ability to maintain a steady growth trajectory. Males appear to lack the robust downstream signaling pathways activated by ERs that drive maturation and provide resilience in females; the male EGP is far more dependent on SIRT1 to maintain a standard growth trajectory.

The different hormonal environments in males (high testosterone, which is partly converted to estrogen) and females (high estrogen and no androgens) likely lead to different regulatory demands on SIRT1. SIRT1 appears to have a different physiological impact depending on the dominant hormonal signals.

In summary, the connection between SIRT1 and estrogen in the EGP appears to be a key regulatory axis for longitudinal bone growth, however as we did not measure sex hormones, receptor signaling, or downstream molecular mediators in the EGP, it should be noted that the interaction between SIRT1 and sex hormones is only one or several possible mechanisms. Both male and female CKO mice exhibited marked growth impairment under normal conditions compared to CTL, supporting the important role of SIRT1 in promoting chondrogenesis and maintaining cellular homeostasis. However, while male showed hyper-responsiveness to refeeding, females CKO showed only moderate alterations, this may be due to the presence of additional pathways important for growth. SIRT1 in the HZ is needed for optimal CU growth and regaining bone quantity/quality following nutritional deprivation, for both sexes. Its absence constrains the anabolic response to refeeding, leading to persistent deficits even after nutrition is restored.

Immunohistochemistry performed using SIRT1 antibody in the EGP of growing mice showed a significant reduction in the level of Sirt1 protein. We acknowledge that although we demonstrated that ColX-Cre drives Sirt1 recombination in cartilage from aged patellae, we did not directly confirm excision of Sirt1 in hypertrophic chondrocytes of juvenile long-bone EGPs at the specific ages studied. This was due to the limited availability of tissue at these developmental stages and the extremely small volume of the EGP.

Another limitation of the study is that we did not measure sex hormones, receptor signaling, or downstream molecular mediators in the EGP, such as Wnt/β-catenin pathway that SIRT1is known to interact with. As mechanistic insight is very problematic to show *in vivo*, in a consecutive study we are using an *in vitro* system to further analyze this interaction, by removing SIRT1 from ATDC5 cells using CRISPR (paper in writing).

To conclude, we have shown that SIRT1 is a critical protein for the proper process of endochondral ossification, Sirt1 deletion in hypertrophic chondrocytes is associated with differences in bone length and microarchitecture, with sex-dependent patterns, in as yet unresolved mechanism. SIRT1 in the HZ is essential for full longitudinal growth, proper bone mass acquisition, and the adaptive response to nutritional fluctuations. SIRT1 loss dampens both the steady-state bone status and the anabolic, CU potential of the skeleton following nutritional stress, underscoring its significance in both baseline and challenged conditions for longitudinal and cortical bone health. SIRT1’s role makes it a potential target for therapeutic strategies aimed at modulating growth and addressing growth-related disorders, with emphasis on sex specificity.

## Data Availability

The raw data supporting the conclusions of this article will be made available by the authors, without undue reservation.

## Supplementary material

Supplementary Figure 1 Anti SIRT1 IHC staining of EGP mice age 25d. **(A)** CTL mice, **(B)** CKO mice. Red staining is considered as a positive.

Supplementary Figure 2 EGPs from mice age 25d were stained with hematoxylin-eosin-alcian blue. **(A)** CTL mice, **(B)** CKO mice.
